# Determining Plasma Protein Variation Parameters as a Prerequisite for Biomarker Studies—A TMT-Based LC-MSMS Proteome Investigation

**DOI:** 10.3390/proteomes9040047

**Published:** 2021-12-01

**Authors:** Lou-Ann C. Andersen, Nicolai Bjødstrup Palstrøm, Axel Diederichsen, Jes Sanddal Lindholt, Lars Melholt Rasmussen, Hans Christian Beck

**Affiliations:** 1 Department of Ophthalmology, Lillebaelt Hospital, DK-7100 Vejle, Denmark; Lou-Ann.C.Andersen@rsyd.dk; 2 Department of Clinical Biochemistry and Pharmacology, Odense University Hospital, DK-5000 Odense, Denmark; Nicolai.Bjodstrup.Palstrom@rsyd.dk (N.B.P.); lars.melholt.rasmussen@rsyd.dk (L.M.R.); 3 Center for Clinical Proteomics (CCP), Odense University Hospital, DK-5000 Odense, Denmark; 4 Center for Individualized Medicine in Arterial Diseases (CIMA), Odense University Hospital, DK-5000 Odense, Denmark; Axel.Diederichsen@rsyd.dk (A.D.); Jes.Sanddal.Lindholt@rsyd.dk (J.S.L.); 5 Department of Cardiology, Odense University Hospital, DK-5000 Odense, Denmark; 6 Department of Cardiothoracic and Vascular Surgery, Odense University Hospital, DK-5000 Odense, Denmark

**Keywords:** inter-individual biological variation, plasma proteins, plasma proteomics, power analysis, sample size determination

## Abstract

Specific plasma proteins serve as valuable markers for various diseases and are in many cases routinely measured in clinical laboratories by fully automated systems. For safe diagnostics and monitoring using these markers, it is important to ensure an analytical quality in line with clinical needs. For this purpose, information on the analytical and the biological variation of the measured plasma protein, also in the context of the discovery and validation of novel, disease protein biomarkers, is important, particularly in relation to for sample size calculations in clinical studies. Nevertheless, information on the biological variation of the majority of medium-to-high abundant plasma proteins is largely absent. In this study, we hypothesized that it is possible to generate data on inter-individual biological variation in combination with analytical variation of several hundred abundant plasma proteins, by applying LC-MS/MS in combination with relative quantification using isobaric tagging (10-plex TMT-labeling) to plasma samples. Using this analytical proteomic approach, we analyzed 42 plasma samples prepared in doublets, and estimated the technical, inter-individual biological, and total variation of 265 of the most abundant proteins present in human plasma thereby creating the prerequisites for power analysis and sample size determination in future clinical proteomics studies. Our results demonstrated that only five samples per group may provide sufficient statistical power for most of the analyzed proteins if relative changes in abundances >1.5-fold are expected. Seventeen of the measured proteins are present in the European Federation of Clinical Chemistry and Laboratory Medicine (EFLM) Biological Variation Database, and demonstrated remarkably similar biological CV’s to the corresponding CV’s listed in the EFLM database suggesting that the generated proteomic determined variation knowledge is useful for large-scale determination of plasma protein variations.

## 1. Introduction

Information on intra-, inter-individual, and analytical variation for measures of components in plasma is important for various reasons, for example for sample size calculations in clinical experiments, the evaluation of specific analytes as screening-, diagnostic-, or monitoring-marker for disease, and in studies attempting to define the influence of genetic and environmental variations on a specific biochemical component. Moreover, these measures of biological variations are prerequisites for the determination of quality demands before the use of measurements of a particular component in clinical situations. Despite this, information on biological variation of most plasma proteins in humans are almost absent, and also relatively low in sources compiling these data. For example, the European Federation of Clinical Chemistry and laboratory medicine (EFLM) Biological Variation Database contains variation data for less than 20 proteins out of the 200 most abundant proteins present in human plasma [1].

By modern proteome analysis it is, however, possible to identify and quantitate many proteins at the same time using only one sample from each individual. Actually, the recent developments in mass spectrometers in terms of sensitivity, scan speed, and dynamic range have enabled the identification and quantification of hundreds to thousands of proteins in a plasma sample in a single proteomic experiment as recently demonstrated [2,3]. Moreover, this number of proteins analyzed by mass spectrometry-based plasma proteomics is further extended by the introduction of immunoaffinity-based depletions methods [4] and affinity-enrichment methods [5] prior to mass spectrometry analysis.

A few attempts have been made to determine the biological and analytical variation of plasma proteins, either individually or together in groups of functionally related proteins using proteomics technologies. These include for example the analysis of the experimental variation in two-dimensional difference gel electrophoresis (2-DIGE) analysis of human plasma [6], the targeted analysis of Chromosome 18-encoded plasma proteins using selected reaction monitoring [7], and the targeted analysis of groups of plasma proteins that are linked to inflammation and cancer using multiplex immunoassays [8]. Moreover, very recently, the inter-individual variability of more than 200 proteins in dried blood spots was measured by multiple reaction monitoring [9]. While liquid chromatography-tandem mass spectrometry (LC-MS/MS) methods haves been used to study the analytical variability, inter-individual variability, and gender variations of human cerebrospinal fluid- and human urine proteomes [10,11], no attempt has yet been made to determine the inter-individual biological variations and analytical variations of the plasma proteome by quantitative proteomics based on nano-LC-MS/MS analysis.

In this study, we hypothesized that it will be possible to generate inter-individual biological variation and analytical variation data of several hundred abundant plasma proteins by the measurement of plasma samples from 42 individuals using nano-LC-MS/MS and tandem mass tag (TMT)-labeling in a quantitative design. From the data, we will calculate the technical, biological, and total variation of the most abundant plasma proteins measured. Moreover, we will create estimates for power analysis and sample sizes in clinical proteomic studies, and compare variation data with the corresponding values already present in the EFLM database.

## 2. Patients and Methods

### 2.1. Patients and Plasma Samples

Plasma samples obtained from 42 individuals enrolled in The Danish Cardiovascular Screening Trial (DANCAVAS) [12], collected in EDTA tubes, processed for proteome analysis, and used as described below. Plasma samples were prepared and frozen immediately after the blood samples were taken. We selected samples from individuals with low-density lipoprotein (LDL) levels in both the high and the low end. The collected plasma samples were diluted x10 with PBS followed by the determination of the protein concentration using the Pierce BCA protein assay kit (Thermo Scientific, Rochford, IL, USA). Ethical approval was obtained by the Southern Denmark Region Committee on Biomedical Research Ethics (S-20140028). Plasma proteins (100 µg) were acetone precipitated by the addition of 500 µL ice-cold acetone followed by incubation at −20 °C for 1 h and centrifugation (20,000× *g*, 4 °C, 10 min). To ensure near-to-complete dissolution of proteins, the resulting protein pellet was re-dissolved first by the addition of 10 µL of an 8 M urea/0.5 triethylammonium bicarbonate (TEAB) solution and incubation for 10 min in an ultrasonic bath chilled with ice followed by the addition of 90 µL 0.5 M TEAB. Reduction was performed by the addition of 5 mM dithiothreitol (DDT, 50 °C for 30 min), and blocking of the reduced sulfhydryl groups was carried out by incubation with 15 mM iodoacetamide (IAA, 30 min in the darkness at room temperature). Tryptic digestion was performed by the addition of trypsin (Promega, Madison, WI, USA) (protein:trypsin ratio: 50:1 *w*/*w*) and incubation at 37 °C overnight. Clinical characteristics of the patients included in this study are summarized in Table 1.

### 2.2. Stable Isotope Labeling of Protein Samples with 10 Plex Tandem Mass Tags

Ten µg fractions of the tryptic digests were tagged with the 10-plex TMT isobaric labeling kit (Thermo Scientific, Rockford, IL, USA) using standard procedures. The labeling of the plasma samples was carried out as follows. A pool of all samples (internal control) was labelled with TMT label reagent 126 and also with TMT label reagent 131, whereas the 42 patients’ samples were randomly tagged with the TMT reagents 127N, 127C. 128N, 128C, 129N, 129C, 130N, and 130C. Labeling efficiency was checked by searching data with 6-plex TMT tags as variable modification and calculating the proportion of unlabeled peptides as identified in the searching of raw data. Labeling efficiency was >95%. Each set of the labeled samples were pooled in equal ratios, purified using custom-made microcolumns packed with reversed-phased material (equal *w*/*w* amounts of Poros R2 and Oligo R3 material) followed by fractionation into four fractions using reversed phase high-pH liquid chromatography. Briefly, samples were loaded onto an ACQUITY UPLC^®^ M-Class CSH^TM^ C18 column (130 Å, 1.7 µm bead size, 300 µm id × 100 mm length) using a linear gradient from 10% solvent B (20 mM ammonium formate in 80% acetonitrile (ACN), pH 9.3) to 55% solvent using a 25 min linear gradient at 6 µL/min flowrate on a Dionex Ultimate 3000 RSLnano system inline coupled to a Dionex 3000 Ultimate UV detector and a Dionex Ultimate 3000 autosampler configured as a fraction collector (Thermo Scientific, Bremen, Germany).

### 2.3. Nano-LC-MS/MS

Nano-LC-MS/MS analysis of the 44 fractionated samples was conducted on an Orbitrap Eclipse mass spectrometer (Thermo Fisher Scientific, San Jose, CA, USA) equipped with a nano-HPLC interface (Dionex UltiMate 3000 nano-HPLC, Thermo Scientific, Bremen, Germany). The samples (5 µL) were loaded onto a custom made fused capillary pre-column (2 cm length, 360 µm OD, 75 µm ID packed with ReproSil Pur C18 5 µm resin (Dr. Maish, GmbH, Ammerbuch, Germany)) with a flow of 3 µL/min for 8 min. The trapped peptides were separated on a custom made fused capillary column (25 cm length, 360 µm OD, 75 µm ID, packed with ReporSil Pur C13 1.9 µm resin) using a linear gradient ranging from 91 to 86% solution A (0.1% formic acid, Fluka, Seetze, Germany) to 25 to 34% B (80% acetonitrile (J.T. Baker, Gliwice, Poland) in 0.1% formic acid) over 77 min followed by 5 min at 90% B and 5 min at 98% A at a flow rate of 250 nL per minute. Mass spectra were acquired in positive ion mode applying automatic data–dependent switch between an Orbitrap survey MS scan in the mass range of 400 to 1200 *m*/*z* followed by peptide fragmentation applying normalized collisional energy of 40% in a 3-s duty cycle. Target value in the Orbitrap for MS scan was 400,000 ions at a resolution of 120,000 at *m*/*z* 200 and 125,000 ions at a resolution of 50,000 at *m*/*z* 200 for MS/MS scans. Ion selection threshold was set to 50,000 counts and the isolation window was 0.7 Da. Selected sequenced ions were dynamically excluded for 30 s.

### 2.4. Data Analysis

All 44 raw data files (eleven 10-plex TMT sets each fractionated into four fractions) were processed and quantified using Proteome Discoverer version 2.4 (Thermo Scientific, San Jose, CA, USA) integrated with the Sequest search engine. Search criteria were as follows: Protein database: Uniprot Human database (downloaded September 2019, 25,252 entries), trypsin, one missed cleavage allowed, carbamidomethylation at cysteines and 6-plex TMT at lysine and N-terminal amines were set as fixed, while methionine oxidation and deamidation were set as dynamic. Precursor mass tolerance was set to 8 ppm and fragment mass tolerance was set to 0.05 Da. The Percolator node was used to filter out non-confident peptides and FDR was calculated using a decoy database search and only high confidence peptide identifications (False discovery rate < 1%) were included. Protein quantifications were based on a minimum of 1 unique peptide per protein, and TMT reporter ion signals were corrected using correction factors as indicated by the manufacturer in the specific reaction kit no. TC264196 is available at https://www.thermofisher.com/search/results?query=TC264196&focusarea=Search%20All, accessed on 24 November 2021. Normalization was carried out using global equal sum (i.e., normalization sum the summaries of all proteins in each channel, and equalize the sums over all channels and runs). Scaling was carried out on averaged controls (mass tag 126) across all files using the available settings in PD2.4. Peptide abundances relative to the internal control sample (mass tag 126) were then calculated using the normalized and scaled values.

### 2.5. Calculation of the Analytical Variability and Inter-Individual Biological Variability

All 42 plasma samples were prepared individually in duplets giving a total sample quantity of 84 samples. Descriptive statistics (mean, standard deviation, and variation coefficients) were calculated individually for all identified plasma proteins. Plasma proteins were included, if a quantitative readout was available in more than or equal to 50% across all 84 samples. Calculated CV-values of the analyzed plasma proteins were compared with corresponding CV-values for all overlapping proteins also present in the EFLM database.

#### 2.5.1. Analytical Variation

The percentage analytical variation (*CV_analytical_*) for each of the 265 proteins detected in more than or equal to 50% of the 42 duplet samples was defined as the mean of the individual analytical variations (*CV_pt_*):

*CV_analytical_* = mean *CV_pt_* = mean (*SD_pt_*/*Mean_pt_*) × 100,

where *Mean_pt_* is the mean the relative abundance of the 42 double determinations of each of the 265 proteins detected across all 84 plasma samples. *SD_pt_* is the corresponding standard deviations. The pre-analytical variation (*CV_pre_*) was defined to be zero.

#### 2.5.2. Inter-Individual Biological Variation

The percentage inter-individual biological variation (*CV_biological_*) was calculated by using the percentage total variation (*CV_total_*) and *CV_analytical_* in the following equation:

$$CV_{biological}=\sqrt{CV_{total}^{2}-CV_{analytical}^{2}},\;CV_{total}=(SD_{total}/Mean_{total})\times 100,$$

where *Mean_total_* is calculated as the mean of the relative abundances for each of the detected proteins and *SD_total_* is the corresponding standard deviation.

#### 2.5.3. Sample Size Calculation

The sample size was calculated using the following formula [13]:

$$n=\frac{2{\left(Z_{\alpha }-Z_{1-\beta }\right)}^{2}\times \sigma^{2}}{\Delta^{2}},$$

where *n* is the sample size, *Z_α_* is the 2-sided *α*-error (1.96; *p* < 0.05), *Z*_1−*β*_ is the power (0.8416 at 80%), *σ* is the standard deviation for each protein, and Δ is the effect size (the protein relative abundance between two groups).

## 3. Results

### 3.1. The Proteome Dataset

In the present study, 42 plasma samples from 42 individuals (41 men) made available to the present study from the DANCAVAS trial with LDL-values ranging from normal to high levels were used for the determination of inter-individual biological variation of plasma proteins by nano-LCMSMS-based proteomics. The samples were processed individually in duplets as described in the methods sections, and the resulting 84 samples were analyzed by nano-LC-MSMS in a 10-plex TMT setup. We retrieved a total of 421 medium-to-high-abundant plasma proteins whereof 265 proteins were present in more than or equal to 50% of all 84 samples. The plasma proteins were relatively quantified against an internal plasma control sample (a pool of all samples) and used for coefficients of variation calculations. The analytical CV for each of the analyzed proteins was calculated as the mean of the *SD_pt_* of 42 double determinations divided by the mean of the corresponding relative abundances. The inter-individual biological variation was determined as the square root of the difference between the squared total variation and the analytical variation. Both methods are commonly used for CV calculations in clinical biochemistry laboratories. Sample size determination is a pivotal aspect of any clinical biomarker study to ensure that the experimental design has sufficient power to detect changes in protein abundances with statistical significance, so we used the determined variance data for sample size estimation at different changes in protein abundances.

### 3.2. Analytical Precision of the LC-MSMS Method and Inter Individual Variation of 265 Plasma Proteins

**Figure 1:**
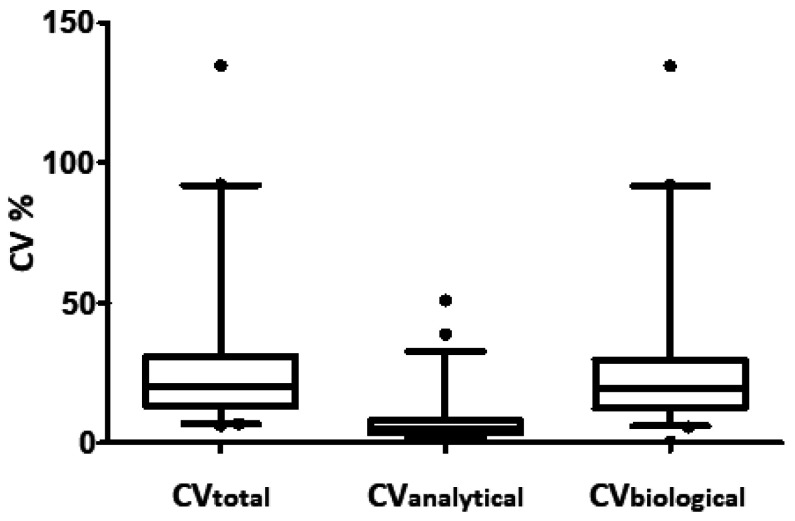
Boxplots showing median values and 1–99 percentiles for the total variation (*CV_total_*), analytical variation (*CV_analytical_*), and inter-individual biological variation (*CV_biological_*) as determined for 265 plasma proteins by quantitative proteomics (Supplementary Table S1).

**Figure 2:**
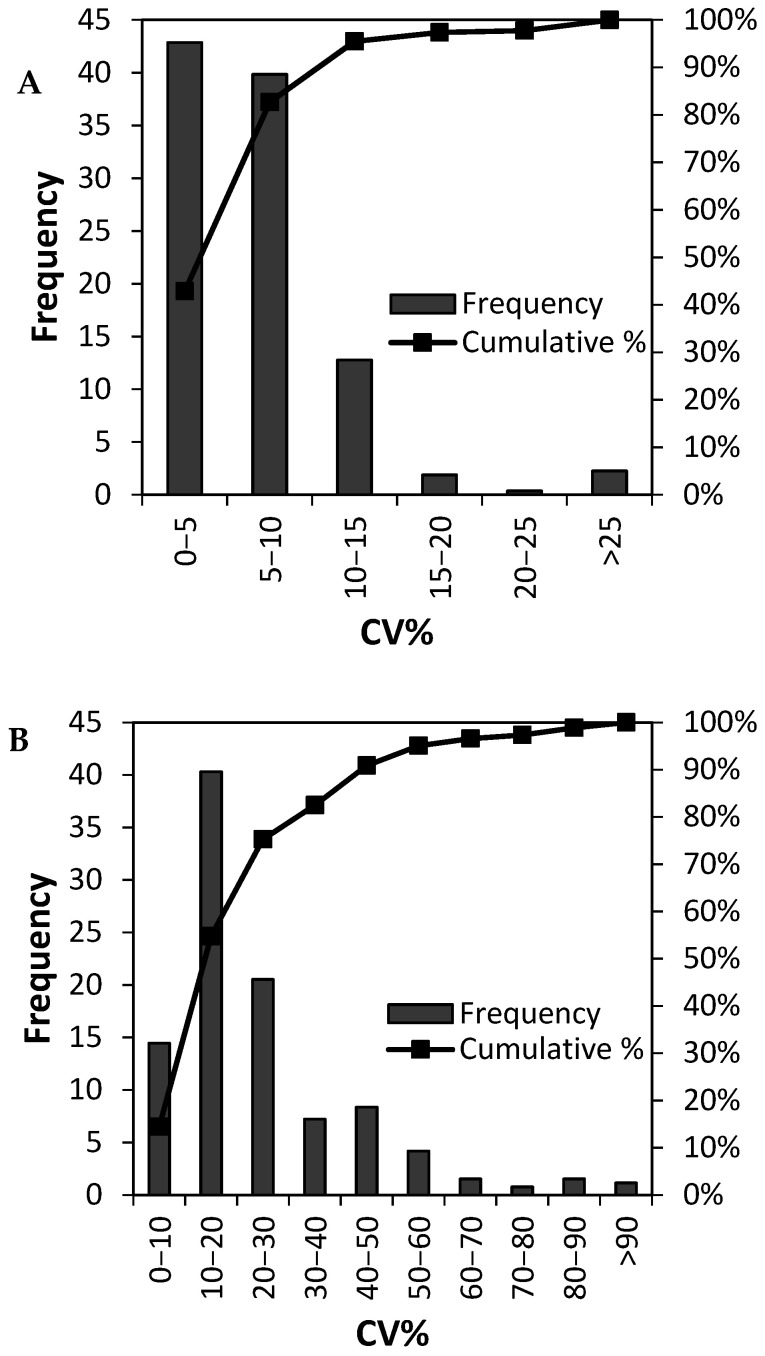

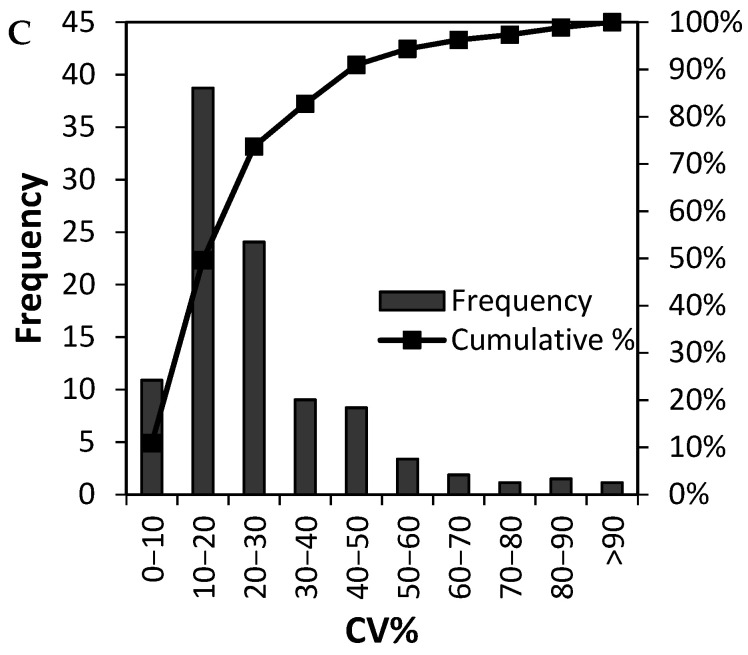
Distribution and frequency of protein coefficients of variations for (**A**) the technical variations, (**B**) biological variations, and (**C**) total variations for the 265 measured proteins.

We determined the total variation (*CV_total_*), analytical variability (*CV_analytical_*), and inter-individual biological variation (*CV_biological_*) for the 265 plasma proteins included in the calculations as described in the methods section (Figure 1 and Supplementary Table S1). The median *CV_total_* of all 265 proteins was determined to be 20.1% (Figure 1) whereas the median inter-individual biological variation, *CV_biological_*, was 19.2%. As expected, the median *CV_analytical_* was markedly lower and determined to be 5.3%. Figure 2 displays the histogram of CV % for the analytical variation (Figure 2A), inter-individual variation (Figure 2B), and total variation (Figure 2C), and the corresponding cumulative number of proteins. For the analytical CV %, more than 225 out of 265 measured proteins showed a CV of less than 10% (Figure 2A). By contrast, less than 20 proteins displayed an inter-individual biological variation (Figure 2B) and total variation (Figure 2C) of less than 10%. The total variation was slightly higher than the inter-individual variation that again was markedly higher than the analytical variation. Considering the technical variation alone and the total variation (the combined technical and inter-individual variations) illustrates that there is a significant biological variation that should be considered when determining the appropriate experimental sample size.

### 3.3. Sample Size Determination

A pivotal aspect of the planning of any clinical biomarker study is the calculation of the sample sizes to determine the differences between two groups. We used the experimentally determined standard deviations for sample size estimation. Sample sizes were calculated using α = 0.05 and 1 − β = 0.8, commonly chosen values for significance and power analysis. As examples, the investigated values for effect sizes (relative changes in protein abundance) were taken as 1.1, 1.2, 1.5, and 2.0, and the required samples sizes were calculated as described in the methods section. Results are summarized in Table 2. Clearly, the variation in protein quantities has a tremendous effect on the number of individuals required in each group or cohort to sufficiently power a study. For example, an experiment with a minimum required power of 0.80, 33 individuals were required to consider a 1.2-fold change to be significant for proteins with variances within the 70th percentile (70% least variant proteins). This number increases to 58 individuals when including proteins with variance up to 80%, and rises dramatically to 712 individuals to cover all proteins. By contrast, only three individuals are required in each group to detect a 2-fold change with a power of 0.8 for 85% of proteins (85% least variant).

### 3.4. Can Biological Variation of Plasma Protein Be Determined by TMT-Based Relative Quantification

Biological variation data are primarily used to aid in diagnosing and monitoring disease, and are traditionally generated by measuring a single component in a well-defined experimental setup using standardized laboratory methods [15] such as enzyme-linked immunosorbent assays (ELISA). To test the validity of the TMT-based relation quantification method for plasma proteomics we compared the calculated CV-values with corresponding values from the EFLM database revealed an overlap of only 17 plasma proteins out of the 265 plasma proteins present in our dataset. CV-values for these 17 proteins found in both the EFLM database and our dataset are summarized in Table 3 that shows the coefficients of variation (*CV_total_*, *CV_analytical_* and *CV_biological_*) for 17 plasma proteins from the present study, and the corresponding inter-individual biological CVs (*CV_biological_*) from the EFLM Biological Variation Database. Interestingly, the biological CVs estimated in our study show for the vast majority of the 17 proteins listed a remarkable similarity with the corresponding values from the EFLM database.

## 4. Discussion

In this study, we investigated parameters that are important to consider when designing large-scale TMT-experiments for plasma biomarker discovery in a realistic sample cohort. We determined the analytical variation of a TMT-based quantitative plasma proteomics workflow together with the inter-individual variation and the total variation of the relative abundance of the measured plasma proteins.

Plasma samples from a 42-patient cohort with varying LDL-levels were analyzed in doublets in a quantitative manner by using nano-LC-MS/MS combined with 10-plex TMT isobaric tagging. We quantitatively measured 421 of the most abundant plasma proteins whereof 265 proteins gave a quantitative readout in more than or equal to 50% of the 84 samples (doublet analysis) measured, and for these coefficient of the variations were calculated.

The results showed that despite there being several pre-analytical steps in our TMT-based workflow from the isolation of proteins over digestion, purification, and isobaric labelling steps to MS analysis, the median variance of the technical process was estimated to be 5.3%. Actually, more than 95% of the analyzed proteins (253 out of 265) showed a technical variance of less than 15%, which is an accepted analytical variance for many biochemical assays routinely used in clinical chemistry laboratories [15]. By contrast, the combined technical and biological variation (the total variation) showed a significantly higher median variance (20.1%) clearly illustrating that there is a significant biological variation that needs to be considered when determining the appropriate sample size for a given TMT-based proteomic experiment. As expected, we determined the median biological variation to be slightly lower than the median total variance.

In contrast to technical variation, the biological variation is protein, patient, and disease dependent. Thus, the chosen sample size of a given TMT-based clinical proteomic experiment should take these variables into account. Sample size calculation can be carried out using a power analysis. The power of a given experiment depends on the variance in protein expression, number of replicates, and the required significance level. Common choices for significance and power analysis for designing clinical trials are α = 0.05 and β = 0.8. Using these parameters at effect sizes (change in protein abundance) of 1.5 and 2, which are commonly chosen fold change cut-offs in proteomics studies, the number of samples required to measure a significant change in protein abundance can be calculated, and we found that the required sample size at the 75th variance percentile (inclusion of 75% least variant proteins) was seven and two, respectively. When including all measured proteins, the required patient numbers increased to 114 for a 1.5-fold change in protein abundance and 28 for a 2-fold change in protein abundance. These values are somewhat lower than the values obtained by Zhou et al. [16] that developed a plasma biomarker discovery workflow based on 8-plex iTRAQ labeling, two-dimensional reversed-phase chromatographic fractionation followed by MALDI TOF/TOF mass spectrometry. They found that six patient samples were required to detect a 2-fold change in protein abundance at the 75th variance percentile with a power of 0.8 and 338 samples were required if all the analyzed proteins irrespective of variance are included in the calculation, and indicate a higher technical variation for the MALDI-TOF/TOF-method compared to our method. Actually, only 37% of the measured plasma proteins in the study by Zhou et al. showed a technical variance of 10% or lower [16], whereas the median technical variance for all proteins measured in our study was 5.3%, and the majority of proteins measured fell within a 10% technical variation.

Mass spectrometry-based proteomics has a great advantage over the conventional immunochemistry methods normally applied in clinical biochemistry in having the capability to measure (in ideal situations) all proteins in a given sample in a single analytical run, and we posed the question of whether our proteomic method has the potential to estimate the biological variation of the large number of proteins present in human plasma. We assessed the total variation (*CV_total_*), analytical variability (*CV_analytical_*), and inter-individual biological variation (*CV_biological_*) for the 265 plasma proteins that were detected across all 42 duplet plasma samples, and compared with inter-individual biological CVs extracted from the EFLM biological variation database. Surprisingly, only 17 plasma proteins out of the 265 measured ones (Table 3, Supplementary Table S1) were present in this database, indicating the literature is scarce on information on the biological variation of plasma proteins in general, and specifically on abundant plasma proteins potentially relevant in a clinical biochemical context. Also worth noticing is that the biological variations estimated by our LC-MS/MS approach were for the majority of proteins remarkably similar to the values extracted from the EFLM biological variation database. For example, our estimate for the *CV_biological_* for CRP was very similar to the *CV_biological_* from the EFLM database (92.3% vs. 87.7%) and in line with previously reported biological CV’s for CRP [17,18], despite that *CV_biological_* values for CRP should be interpreted with some precautions. CRP is an acute-phase protein with low and stable plasma concentrations (around 0.5 mg/mL) in basal conditions in many individuals [19], but may rise in concentration with a factor of 10 to 20 upon an even small inflammation-causing event such as infection or may be present at persistently increased concentrations in plasma from individuals with long-term risk for coronary heart disease [20,21].

### Limitations

The 42 individuals included in the present study were chosen based on lipid profile resulting in a study group that is biased in age and health status (Table 1), which may affect the levels of certain plasma proteins including CRP as mentioned above. Moreover, specific plasma proteins may also display a gender-specific inter-individual variation [11], and as our study group included almost solely males, this may have affected our results. Sample proteolysis may also have affected our results. In order to avoid this, plasma samples were prepared and frozen immediately after collection of blood samples. Moreover, previous studies have shown that plasma proteins are remarkably stable to pre-analytical variables such as prolonged storage at even room temperature when analyzed at the peptide level [22], so we are confident that this effect is reduced to a minimum in our study. Moreover, this is a bottom-up study and therefore does not effectively reflect the native complexity of the proteome since all information concerning critical proteoforms is lost due to the nature of the applied analytical method (i.e., the different protein forms produced from the genome with a tremendous variety of sequence variations, splice isoforms, and the enormous number of different posttranslational modifications) [23]. Finally, our results rely on relative protein quantification using isobaric tags, a method that is known to underestimate the measurement of differences in relative protein concentrations in bottom-up proteomics experiments, mainly due to interference from contaminating peptides that are co-isolated together with the target peptide [24].

## 5. Conclusions

In conclusion, study (1) generated inter-individual biological variation and analytical variation data for 265 abundant plasma proteins, using LC-MS/MS to un-depleted plasma samples and isobaric tagging in a quantitative design; (2) performed power analysis that provides guidance for future TMT-based clinical proteomics studies; and (3) manufactured a catalogue of the calculated total variation, analytical variability, and inter-individual biological variation of all of the 265 plasma proteins, all of which are useful as a reference work for future biomarker studies and for determining the number of human plasma samples needed for proteomic characterization.

## Figures and Tables

**Table 1:** Relevant clinical characteristics of the patients involved in the study.

Number of Patients (*n*)	42
Age range (mean)	64–74 (68)
BMI range (mean)	21.5–42.1 (28.2)
Male sex (%)	41 (97.6)
CRP range (mg/L) (mean)	0.6–98 (6.8)
LDL range (mmol/L) (mean)	0.4–7.3 (4.6)
HDL range (mmol/L) (mean)	0.8–2.6 (1.2)

**Table 2:** Estimated sample sizes required per group for effect sizes (relative changes in protein abundances) and corresponding confidence intervals [14] from 1.1 to 2.0 at the 70th, 75th, 80th, and 85th variance percentiles (proteins with variance equal to or lower than the specified percentile), and the required sample size at maximum variance (i.e., when including all protein variances in the estimations). For sample size calculations we applied common values for significance and power used in proteomics clinical trial design (α = 0.05 and β = 0.8).

Effect Size	Variance (Percentile)				
	70th	75th	80th	85th	Maximum
1.1	131 (89–213)	177 (120–288)	233 (158–379)	314 (213–511)	2848 (1928–4630)
1.2	33 (22–53)	44 (30–72)	58 (39–95)	79 (53–128)	712 (482–1158)
1.5	5 (4–9)	7 (5–12)	9 (6–15)	13 (9–20)	114 (77–185)
2.0	1 (1–2)	2 (1–3)	2 (2–4)	3 (2–5)	28 (19–46)

**Table 3:** Coefficients of Variation (*CV_total_*, *CV_analytical_*, and *CV_biological_*) for 17 plasma proteins out of the 265 quantified plasma proteins determined by quantitative proteomics and the corresponding inter-individual biological Coefficients of Variation from meta-analysis extracted from the European Federation of Clinical Chemistry and Laboratory Medicine (EFLM) Biological Variation Database [1]. The EFLM database did not contain any CV-values for the remaining 248 proteins analyzed by quantitative proteomics.

Uniprot Accession	Description	*CV_total_* %	*CV_analytical_* %	*CV_biological_* %	EFLM Biological Variation
P17936	Insulin-like growth factor-binding protein 3	15.0	4.0	14.4	0.003 *
P02647	Apolipoprotein A-I	13.0	2.3	12.8	11.2
P01034	Cystatin-C	16.3	6.6	14.9	12.1
P05543	Thyroxine-binding globulin	9.4	3.3	8.8	12.6 *
P01024	Complement C3	10.0	1.8	9.8	15.2
P02766	Transthyretin	18.2	10.6	14.7	19.1
P04114	Apolipoprotein B-100	23.3	1.7	23.2	20.2
P19652	Alpha-1-acid glycoprotein 2	37.4	10.7	35.8	24.1
P02763	Alpha-1-acid glycoprotein 1	24.6	6.2	23.8	24.1
P0C0L4	Complement C4-A	25.4	7.6	24.2	24.5
P0C0L5	Complement C4-B	23.8	11.2	21.0	24.5
P04278	Sex hormone-binding globulin	22.1	6.2	21.2	35.6
P00738	Haptoglobin	38.3	2.7	38.2	39.0
P02768	Serum albumin	7.1	1.9	6.8	5.1
Q15848	Adiponectin	23.9	12.6	20.3	51.2
P01009	Alpha-1-antitrypsin	13.7	2.8	13.4	10.5
P02741	C-reactive protein	92.3	7.6	92.0	87.7

* non-meta studies, all other values are based on metanalysis of biological variation studies.

## Data Availability

The datasets generated and/or analyzed during the current study are not publicly available due to hospital guidelines and legislation regarding personal data. Data will be available from the corresponding author on reasonable request and with permission of Odense University Hospital Legal Department.

## Supplementary Materials

The following are available online at https://www.mdpi.com/article/10.3390/proteomes9040047/s1, Table S1: Proteome data.
